# Data-driven group comparisons of eye fixations to dynamic stimuli

**DOI:** 10.1177/17470218211048060

**Published:** 2021-09-29

**Authors:** Tochukwu Onwuegbusi, Frouke Hermens, Todd Hogue

**Affiliations:** School of Psychology, University of Lincoln, Lincoln, UK

**Keywords:** Eye movements, eye tracking, dynamic stimuli, group comparisons, saccade diagnosis

## Abstract

Recent advances in software and hardware have allowed eye tracking to move away from static images to more ecologically relevant video streams. The analysis of eye tracking data for such dynamic stimuli, however, is not without challenges. The frame-by-frame coding of regions of interest (ROIs) is labour-intensive and computer vision techniques to automatically code such ROIs are not yet mainstream, restricting the use of such stimuli. Combined with the more general problem of defining relevant ROIs for video frames, methods are needed that facilitate data analysis. Here, we present a first evaluation of an easy-to-implement data-driven method with the potential to address these issues. To test the new method, we examined the differences in eye movements of self-reported politically left- or right-wing leaning participants to video clips of left- and right-wing politicians. The results show that our method can accurately predict group membership on the basis of eye movement patterns, isolate video clips that best distinguish people on the political left–right spectrum, and reveal the section of each video clip with the largest group differences. Our methodology thereby aids the understanding of group differences in gaze behaviour, and the identification of critical stimuli for follow-up studies or for use in saccade diagnosis.

## Introduction

Eye tracking technology has made great advancements in recent decades, with vast improvements in the sampling rate, spatial accuracy, requirements on head restraints, and options to display a variety of stimuli to research participants (Duchowski, 2007). Many universities and research institutes, but also private businesses, will now have one or more eye trackers to study observers’ eye gaze at high sampling rates, with limited or no head restraint, where observers can be presented with a broad range of stimuli (simple shapes, photographs, and videos). Great advancements have also been made in computing power and generic analysis packages, meaning that large data sets across many observers and stimuli can now be analysed. Improved eye tracking capabilities have led researchers to move towards more ecologically valid stimuli (Kingstone, 2009), involving photographs (Birmingham et al., 2009; Brockmole & Henderson, 2005; Henderson et al., 2007) and video clips (Shen & Itti, 2012), or even active navigation (Foulsham et al., 2011; Hayhoe & Ballard, 2005). The question, however, arises, how to best analyse the recorded eye movements.

### General eye movement properties

One approach when analysing eye movements in response to visual stimuli is by comparing general eye movement properties, such as fixation durations, saccade amplitudes, and the overall number of fixations and saccades. By comparing such properties across groups or under different task constraints, the effects of, for example, neurological conditions or task on eye movements are investigated. A possible limitation of such an approach, however, is that such general eye movement properties are often difficult to interpret. For example, it is not always clear whether experts (e.g., in surgery, Hermens et al., 2013) are expected to have longer fixation durations on certain regions than novices. Longer fixation durations may be expected if they explore a smaller section of the scene for longer, but longer fixation durations may also indicate that they wait longer before planning their next eye movement, making the interpretation difficult.

### Regions of interest (ROIs)

Another common approach to analysing eye movement data for natural scenes and videos is the use of ROIs, where regions are defined around areas in the scene or video frames that represent objects that may be important for viewers. For example, when interested in how other people’s social cues influence an observer’s eye movements, regions around the eyes, head, body, and arms can be defined, and the properties of fixations on each of these regions can be analysed (e.g., Birmingham et al., 2009).

The ROI approach is feasible when the ROIs are well defined (as in the use of social scenes to study social attention) and when a limited set of static images are used (because of the time involved in manually delineating the ROIs, or in the development of computer vision techniques that may aid in such coding, which often still require review by human observers). Optimal conditions, however, are not always met. It may be unclear what are the possible ROIs. For example, when trying to compare expert and novice surgeons who are looking at laparoscopic images, it may not be directly clear what parts of the images signal differences between the two groups.

A further possible limitation of the ROI approach is that it may not always be the most powerful method to uncover group differences. Comparisons strongly depend on what the researcher considers to be important areas in the image(s) for the group distinction, which restricts the analysis to the ROIs considered. As a consequence, interesting group differences that occur for other parts of the stimuli may be missed, which is particularly a problem in domains where it is less clear what the areas of interest are (e.g., crime scenes, laparoscopic surgery videos, explicit videos).

A further limitation is the highly labour-intensive nature of the approach, requiring the manual coding of ROIs, which is particularly a problem when video clips are used instead of static images, where ROIs need to be coded for every single frame of the video. This, in turn, could lead researchers to decide to subsample the data to restrict the amount of work in the analysis. Even when modern computer vision methods, such as YOLO or RetinaNet, are employed, there is the issue of either finding relevant network weights for such techniques (e.g., ones that will detect people in a scene) or finding sufficient data to train new networks (e.g., for regions not commonly coded, such as body parts in explicit videos or surgical instruments and anatomical structures in surgical videos). Such methods also require some knowledge of computer programming and machine learning, and the result is likely to require review from a human observer.

### Saliency models

Another commonly adopted method is the use of saliency models (Carmi & Itti, 2006; Itti, 2005; Itti & Baldi, 2005). Saliency models make assumptions about the visual system and use these to generate predictions where observers are likely to attend. An important advantage of saliency models over the ROI approach is that regions are defined by a generic model of what parts of an image are likely to be of importance to viewers, not based on expectations of the researcher (Carmi & Itti, 2006; Itti, 2005; Itti & Baldi, 2005). Saliency models, however, focus strongly on low-level features and may therefore be expected to predict similar distributions of attention across groups of attention. Moreover, it has become clear that there are limitations to what patterns of eye movements saliency models can explain (Foulsham & Underwood, 2008; Henderson et al., 2007).

### IMap

While saliency models highlight regions in images that are likely to be attended, they provide no direct means to compare distributions of fixations across groups. One method that provides such group comparisons is iMap (Caldara & Miellet, 2011; Loschky et al., 2015). The method makes use of gaze heatmaps to represent the probabilistic spatial distribution of raw gaze points, which are then compared across conditions or groups to statistically confirm qualitatively observable changes over time (e.g., tightening of gaze clusters) or to detect differences between conditions.

Various studies have used the iMap method to assess differences in eye movements between groups or viewing conditions. For example, Caldara and Miellet (2011) used the iMap method to generate statistical fixation maps to summarise viewing behaviour for images to isolate fixation clusters. Le Meur and Baccino (2013) used the iMap method to assess interobserver variability by determining the natural dispersion of fixations between observers watching the same stimuli. Blais et al. (2008) used iMap to compute the statistical significance of group differences.

The iMap method, however, has some possible limitations. For example, as Eckhardt et al. (2013) argued, regions with significant differences between conditions can be scattered across the stimuli and hard to interpret. Another possible limitation is that iMap uses the same Gaussian width for all fixations, which may pose issues for ROIs of different sizes. Furthermore, although extensive documentation is available, the use of iMap may be nontrivial for less tech-savvy researchers, or those without experience with MATLAB. Finally, the method appears to be computationally expensive, which may limit its use for dynamic stimuli (videos). That said, the power of the method is its use in testing the statistical differences in viewing patterns that do not require assumptions about what regions in an image may be important for viewers.

### Scanpath comparison methods

Other approaches to compare viewing patterns between groups and viewing conditions include the normalised scanpath saliency (NSS), Kullback–Leibler (KL) divergence, Gaussian mixture modelling, and receiver operating characteristics (see Le Meur & Baccino, 2013, for a review). For example, Loschky et al. (2015) used the *z*-normalised gaze similarity, which uses inferential statistics to identify moments in time when the gaze distributions between two groups differ, inspired by the normalised scanpath saliency (NSS) first proposed by Peters et al. (2005).^1^

The method involves a series of processing steps: (1) Interobserver similarity is computed with a leave-one-out procedure whereby a probability map is created by plotting two-dimensional (2D) circular Gaussians around the gaze locations within a specific time window for all but one participant within a condition; (2) the resulting Gaussians are summed and normalised relative to the mean and *SD* of these values across the entire video, *z*-score similarity = (raw values – mean) / *SD*; and (3) the gaze location of the remaining participant is then sampled from this distribution (i.e., a *z*-score is calculated for this participant) to identify how their gaze fits within the distribution at that moment. The resulting *z*-scored values (referred to as gaze similarity) express both (1) how each individual gaze location fits within the group at that moment and (2) how the average gaze similarity across all participants at that moment differs from other times in the video: A *z*-score close to zero indicates average synchrony, negative values indicate less synchrony than the mean (i.e., more variance), and positive values indicate more synchrony. Loschky et al. (2015) utilised this method to compare attentional synchrony between viewing conditions (context vs. no-context) and found that viewers’ eye movements reflect strong attentional synchrony in both conditions compared with a chance level baseline, but smaller differences between conditions.

The KL divergence quantifies the overall dissimilarity between two probability density functions and varies in the range of zero to infinity with zero value indicating that the two probability density functions are strictly equal (Le Meur & Baccino, 2013). Tatler et al. (2005) used the KL divergence to estimate differences in probability distribution of fixation locations for individual observers. However, Tatler et al. (2005) were unable to generate statistical fixation maps for single conditions (and their comparisons) because KL only reports a single index for each comparison. Because KL divergence is not symmetric, it cannot be used to measure distance between two distributions. As a result, it is difficult to localise significant differences between conditions within the stimulus space. As with iMap, less technically skilled researchers may have difficulties employing the method. Likewise, the method may be computationally expensive, which may hamper its use for dynamic stimuli.

### Other approaches

A range of other methods have been developed, for example, to determine to which extent observers are drawn towards the centre or surround of the scene (Tseng et al., 2009) or to determine the variability of eye-gaze patterns across observers (Berg et al., 2009; Dorr et al., 2010; Hasson et al., 2008). Others have compared eye movements with different edited versions of the same video clip, so that the ROIs are defined to see how the editing of the video affects eye movements (Cristino & Baddeley, 2009; Marius’t Hart et al., 2009). Although these methods reveal interesting aspects of the eye movement data, they cannot be directly used to detect group differences.

### Present study

The discussion above has shown that there are several methods to study patterns in eye movements, some of which can be used to compare distributions of gaze fixations across groups. The methods, however, generally have a range of limitations, including (1) they often work best for images, (2) they may involve complex calculations or software that may be difficult to use, and (3) they may involve assumptions about regions that are important in the stimuli or how the visual system operates.

The specific aim of this study is to introduce and test a new, simple method to counteract some of these issues. The general aim of the new method will be to uncover and understand group differences in eye movement patterns, for example, for diagnostics (Benson et al., 2012), skill assessment (Hermens et al., 2013), or to compare offenders and nonoffenders (Fromberger et al., 2012; Hall et al., 2014). The method, however, can also be used to examine differences in eye movement patterns under different conditions (as long as the same visual stimuli are used). The method focuses on the use of dynamic stimuli (videos) and aims to isolate (sections of) the videos that may inform group differences.

#### Proposed method

The proposed method adopts some aspects of the strategy employed by Khan et al. (2012) to compare eye movements patterns in expert and novice surgeons watching a recorded head-mounted video stream of an expert surgeon performing a surgical procedure. Eye movements of both expert groups were compared with the eye movements of an expert surgeon performing the surgery by computing the proportion of frames where the gaze position of the observer was within a set distance from that of the actor. A larger percentage of samples with overlap was found for experts than for novices, suggesting group differences between expert and novice surgeons in their eye gaze.

We extend this method with a frame-by-frame analysis of group differences in viewing patterns so that it is possible to determine which videos and which sections of videos reveal group differences best. Selection of videos can shorten the testing time needed when using videos to classify observers into different groups (e.g., patients and controls) and selection of relevant frames can improve classification.

To make the method easy to adopt by a broad range of possible users, we make use of a standard statistical test to “test” for group differences, namely, Student’s *t*-test. It should be stressed that we only use this test to uncover possible sections of videos that may be relevant to group differences rather than to make statements about whether such differences are statistically significant (which would require corrections for the number of comparisons, which can be large when employed on a frame-by-frame basis). The use of Student’s *t*-test means that the method can be implemented in almost any modern programming language without requiring extensive programming experience (we tried the method with MATLAB, R and Python, but Excel or SPSS may also be an option) and without requiring complex code.

We identify two methods to quantify group differences: (1) separate comparisons of horizontal and vertical gaze position (comparing central tendency differences between groups—either horizontally or vertically, or both) and (2) comparisons of the distance to the group centres (comparing divergence difference between groups). The first method will detect variances in the central tendency of the position of the two groups (in horizontal, vertical, or combined horizontal and vertical directions)—for example, detecting that one group may be focusing on the politician, and the other group on the text at the bottom of the image. The second method may detect whether one group avoids looking at parts of the image. It may, for example, show that one group looks at the politician, whereas the other group may try not to look at the politician (but may still, on average, look at the position of the politician in the image). The latter method may therefore be useful to detect nonsystematic avoidance gaze behaviour (e.g., in applications where participants try to avoid a diagnosis).

For both methods, the following processing steps are involved: (1) the horizontal and vertical gaze position is identified for a particular video frame, (2) gaze positions for each frame are compared between groups with Student’s *t*-tests (either by comparing horizontal and vertical positions, or comparing the distance to the group centre), and (3) videos and sections of videos are identified with large group differences.

#### Validation

We complement these processing steps with a validation step, which tests how well the selected videos and selected frames distinguish between the two groups. This validation makes use of machine learning techniques, where we test whether with the selected videos and frames, a hold-out sample can be classified on the basis of eye-gaze patterns of the remaining participants (thereby mimicking the classification of unseen data, as would be common in saccade diagnostics).

In the main text, we focus on the method that detects group differences in the gaze distance to the group centres (examining possible gaze avoidance behaviour). In the Supplemental Material, we will show that the method that examines position differences between groups (horizontal, vertical, or combined horizontal and vertical) yields slightly worse group membership prediction, but still a prediction well above chance level.

The method that we are developing will ultimately serve clinically relevant comparisons, for example, of samples of sex offenders and nonoffenders, gamblers and nongamblers, people with an eating disorder and controls, or to test for expertise effects, for example, in expert and novice surgeons. For development of the method, employing such groups directly, however, raises ethical concerns as well as practical ones. If our study would reveal that the method does not work, valuable time of vulnerable (patients, gamblers, offenders) or busy (expert surgeon) participants would have been wasted. Recruiting and testing a sufficiently large sample of such groups of participants may also be an issue.

We therefore validate our method in a sample of psychology students and examine whether it can reveal group differences in political views. Reasons for choosing this particular domain were (1) our past experience with measuring people’s political view (Harper & Hogue, 2019), (2) the strong popular interest in political views in an area of increasing polarisation of Western societies (Inglehart & Norris, 2016; O’Hagen, 2016; Taggart & Szczerbiak, 2018), and (3) it not being a domain that has already been extensively studied with eye movements, thereby having the potential to uncover new interesting results.

We focus on classifying participants’ left-right orientation. In Western political systems, a distinction is often made between the left- and right-wing political ideology (Havlík & Stanley, 2015; Katsambekis, 2017; Mudde & Rovira Kaltwasser, 2013). Left-wing ideology champions an inclusive society, describes people on the basis of class, and aims to protect the masses from oppression. Right-wing ideology leads to a more exclusive society and places greater emphasis on tradition and cultural values above everything else (Havlík & Stanley, 2015; Mudde & Rovira Kaltwasser, 2013; Rooduijn & Akkerman, 2017). Importantly, this left-right distinction has been shown to manifest itself in the behaviour of populist parties (Havlík & Stanley, 2015) and voting choice (Jou & Dalton, 2017; Otjes & Louwerse, 2015). The consequences of the left-right distinction are formalised in the Party Representation Model (Jou & Dalton, 2017).

We here test whether we can find differences in viewing patterns of participants who self-identify (on the basis of questionnaires) as affiliated to left-wing or right-wing views. As stimuli, we used a set of short video extracts of left-wing and right-wing politicians in various contexts (e.g., one-to-one interviews, mass rallies) to determine whether the group differences vary across videos.

We used videos of four politicians (Corbyn—United Kingdom, May—United Kingdom, Obama—United States, and Trump—United States) and used three scales to establish whether participants were left-leaning or right-leaning, in addition to asking them for their party affiliation. We here focus our discussion of the results on the two U.K. politicians (as participants were U.K.-based) and the two party splits that showed considerable overlap (based on party affiliation and the Ontological Insecurities Scale [OIS]) to reduce the number of videos in the validation and the number of comparisons between groups.

Instead of a few long video clips of each politician, we chose to use many shorter video clips, showing the same politicians in different contexts. Although we did not have strong a priori expectations regarding the types of video clips that would yield the strongest group differences, we may expect that video clips with people in the background to reveal larger group differences. This is because when just the politician is in view, there may be little else for observers to look at, and consequently, group differences may be small.

## Method

### Participants

Forty-four students from the University of Lincoln (36 females, 18–38 years of age, *M*_age_ = 21, *SD* = 3.9) took part in the study that was approved by the local ethics committee. Twenty-one said to be affiliated to the Labour party while 23 others could be classified as non-Labour (i.e., either a member of the conservative party or were nonmembers). There were no significant differences in the distribution of males and females across the groups.

### Design

Each participant saw the same randomised sequence of the 80 video clips, with a mixture of 20 video clips showing Corbyn (left-wing/Labour, United Kingdom), May (right-wing/Conservatives, United Kingdom), Obama (left-wing/Democrats, United States), and Trump (right-wing/Republicans, United States). To limit the number of features in the various machine learning models and number of data plots, we will focus on the eye tracking data for the two U.K.-based politicians (Corbyn and May). Data for the other two politicians are available on https://osf.io/4ch9q/?

### Stimuli

The 80 video clips were sourced from YouTube and reduced in length using the OpenShot software package. Reducing the length of the videos not only allowed for varying the context in which the politician was shown, but also ensured that we complied with the fair use copyright policy for academic research. Each reduced video clip lasted around 16 s and showed politicians in various contexts (in isolation, one-to-one interview, rallies).

Besides asking participants for their political orientation, three questionnaires were used to establish participants’ political orientation and other demographics. These were (1) a sociodemographic questionnaire (gender, age, nationality, political affiliation), (2) the OIS (Harper & Hogue, 2019) measuring respondents’ subjective feelings of insecurity about “Social Change” and “Systemic Inequality,” (3) the Political Attitudes Scale (PAS; Everett, 2013), and (4) the Right-Wing Authoritarianism Scale (RWAS; Altemeyer, 1988). Party affiliation and the OIS led to similar grouping of participants into left-leaning and right-leaning. The RWAS and the PAS led to different groupings for unclear reasons. To reduce the number of group comparisons to discuss, we here focus on the splits by party affiliation and the OIS. The remainder of the data are available on the OSF archive for the study: https://osf.io/4ch9q/?

### Apparatus

Stimuli were presented on the 24-inch screen of a Tobii T60 XL eye tracker at a 1280 × 900 video resolution and from a distance of around 65 cm, maintained with a chin rest. Eye movements in both eyes were combined into a binocular measure of gaze positions and tracked at a sampling rate of 60 Hz. The Tobii T60 XL has a reported resolution of 0.5° and accuracy of 0.35° and applies both bright and dark pupil tracking. While the eye tracker automatically parses the recorded eye movements into fixations, saccades, and blinks, we used the raw eye movement recordings per video frame (sampled at 30 fps), coding blinks as missing values. The reason is that the alignment of frame-by-frame eye movements between groups is straightforward, whereas alignment of fixations is not due to their different onsets and offsets between participants over time.

### Procedure

Participants were tested individually in a quiet, darkened room. They were asked to take place at a desk looking directly at the screen of the Tobii eye tracker with their chin resting on the chin rest. Before presentation of the stimuli, the default 9-point calibration sequence was performed, involving participants fixating a series of nine red circles distributed across the screen. Calibration was visually inspected by the experimenter who accepted calibration when recorded gaze points overlapped with the positions of the calibration stimuli. Following successful calibration, participants were provided with written instructions on the screen and were afterwards prompted to press a key to begin the experiment. Participants were shown the 80 video clips in succession, whereas their eye movements were recorded, which was done in a single session of around 20 min. After watching all the 80 video clips, they filled out the pen-and-paper questionnaires and were debriefed and thanked for their participation.

### Data analysis

**Figure 1.**
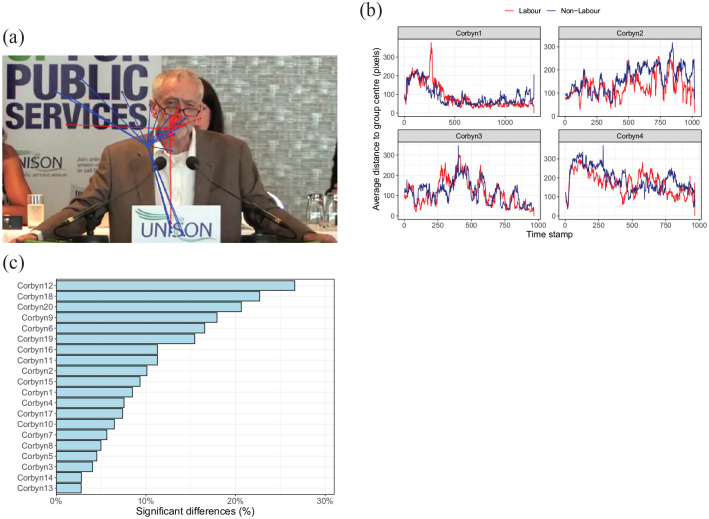
Illustration of the method. (a) Example of a video frame with superimposed the gaze positions of the Labour participants (red dots) and the non-Labour participants (blue dots). Lines connect the gaze position of each participant with their group centre. The method can either compare the two group centres, or the average or summed length of the lines connecting the gaze positions to the group centre. We here focus on the latter. (b) Average distance to group centres over time for Labour and non-Labour participants. Higher values indicate more variance in the gaze position within the group. (c) Percentage of frames with a “significant” difference in the distances to the group centres (example based on videos of Jeremy Corbyn and a split between Labour and non-Labour participants), revealing videos with small and videos with large group differences.

Figure 1 illustrates the method employed for data analysis. For each video frame (sampled at 30 fps), the gaze position for each participant was extracted from the raw eye tracking data (sampled at 60 Hz, meaning that only the first of two samples for each frame was used). We here report the results for one of two methods to isolate group differences, namely, the method using the distance to the group centre (the alternative method, analysing the horizontal and vertical difference separately showed less clear group differences for this set of data).

To isolate video clips with large differences in gaze behaviour between the two groups, we thus computed for each frame, participant, and video combination the average Euclidean distance (in pixels) to each of the group centres, thereby focusing on the dispersion in eye movements inside each group. We then computed the percentage of frames that showed a “significant” difference in these distances to the group centres using a Student’s *t*-test (uncorrected critical *p* value of .05).

### Validation: machine learning

To examine whether observed group differences can be used to classify newly observed participants into Labour or non-Labour leaning based on their eye movements, we used machine learning (classification) algorithms. Because, a priori, it is unclear which machine learning method works best, we tested several methods: a logistic regression, a k-nearest neighbour (KNN), a decision tree, and a random forest classifier. We employed R’s caret package (Breiman, 2001) using the default parameters of the various models. We here present the results based on the distance towards the group centres (possibly reflecting avoidance of stimuli within the image—for example, avoiding looking at Corbyn). Results for predictions on the basis of average horizontal and vertical gaze positions and selection of frames and videos with these gaze positions are shown in the Supplemental Material.

To limit the number of features entered into each model (in machine learning terms, we have relatively few cases—namely, the 44 participants, compared with the number of features—frames sampled at 30 fps), we computed one average distance to the group centre for each combination of participant and video, instead of entering the individual gaze samples (at 60 Hz over 80 videos of about 16 s each). The focus on the U.K. politicians also reduced the number of features in the models.

To examine the effects of (1) selecting videos with large differences, and (2) selecting samples with significant differences, we fitted machine learning models for averages based on (1) all frames from all videos (no selection of videos or frames), (2) all frames from videos with large group differences (a 10% “significant” differences threshold was used), (3) only the “significant” frames from all videos, and (4) only the “significant” frames from videos with large group differences (same 10% threshold). If the method were to be used to “diagnose” political affiliation of people based on their eye movements, the first and third method would require showing all videos to a test participant (taking around 20 min), whereas the second and fourth method would focus on a selection of videos (taking less time).

**Figure 2.**
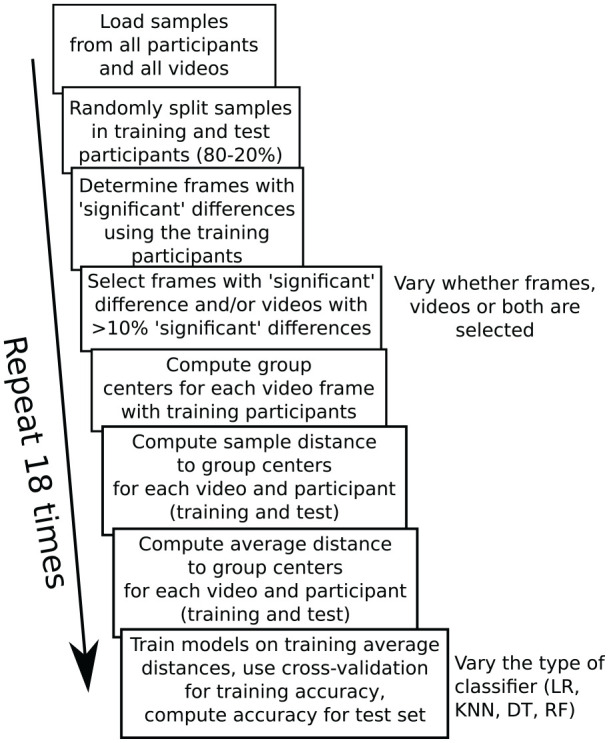
Steps for evaluating how well the proposed method predicts group membership. Throughout the process, training (random selection of 80% of participants) and test (remaining 20% of participants) are kept separately, so that the accuracy on the test participants would reflect performance if a new batch of participants would be classified with the method. The 18 repetitions of the process were used to determine how strongly the end results depend on the random split between training and test participants. The number of repetitions was a balance between computing time and sufficient information about the average performance and variability.

To examine how well new participants (unseen data) would be classified, we split data into a training set (80% of participants) and test set (20% of participants). The test set was set aside, and videos and frames of videos were selected, and machine learning models were trained with the training set. The participants in the test set (yet unseen by the model) were then classified with the trained model to determine how well new participants can be classified on the basis of their eye movements (similar to when the test would be used for diagnostics). The various steps involved in evaluating our method are shown in Figure 2.

Because the number of participants was limited (due to the time involved in testing plus administering the questionnaires) and a single split of the data in a training and test set could reflect the random split of the data to some extent, we relied on multiple random splits of the original data set into training and test sets, and computed the average performance across these multiple random splits. Performance was evaluated for the test set (we used accuracy, as the set was almost perfectly balanced in Labour- and non-Labour participants), and the training set (where we used a fivefold cross-validation) (Bali et al., 2016). Computer code used for the analysis and results from the various processing steps are available from https://osf.io/4ch9q/?

## Results

**Figure 3.**
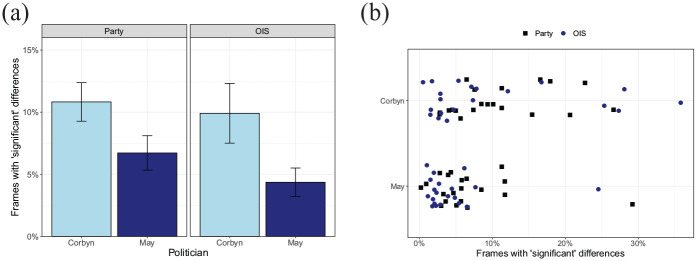
(a) Percentage of frames with a “significant” difference the party split and the OIS split for politicians Corbyn and May. Bars represent standard error of the mean across video clips. (b) Variation in the percentage of frames with “significant” differences across videos and splits.

Figure 3a shows the average percentage of frames with “significant” differences between groups, based on the splits by party affiliation (Labour or non-Labour) and the OIS, separately for the two politicians. Videos of the left-wing politician Corbyn show larger numbers of video frames with “significant” group differences. Highly similar patterns are found for the splits based on party affiliation and the OIS. Figure 3b shows that often the percentage of “significant” frames is around 5%, what can be expected on the basis of chance. Some videos, however, show percentages of frames with “significant” differences of around 30%.

A two-way analysis of variance (ANOVA) testing the effect of split (party or OIS) and politician (Corbyn or May) showed a significant interaction between these two factors, *F*(1, 38) = 10.7, *p* = .036. Within each split, the effect of politician was (marginally) significant, party: *F*(1, 38) = 3.89, *p* = .056; OIS: *F*(1, 38) = 4.35, *p* = .043. Within videos of Corbyn, no significant difference was found between the party and the OIS split, *F*(1, 38) = 0.10, *p* = .75. No difference between splits was found for May videos either, *F*(1, 38) = 1.72, *p* = .20.

### Features of videos with large group differences

To examine whether videos with a larger percentage of “significant” differences have specific features (e.g., more people in the scene, allowing for more variation between participants where to look), we annotated four features of the various videos: (1) setting (rally, television interview, television speech), (2) number of people in the scene (one or more than one), (3) whether text was shown in the display (known to attract attention of viewers; Rayner et al., 2001), and (4) whether the video contained a cut (also known to affect eye movements; for example, Coutrot et al., 2012). In our comparison of the features, we focus on the party split (Labour vs. non-Labour).

**Figure 4.**
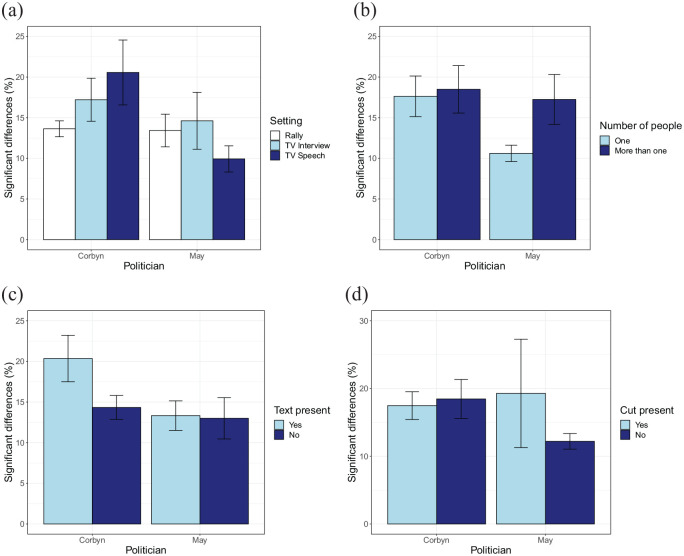
Comparisons examining the effects of features of the videos on the percentage frames with differences, based on the party split. The following features were considered: (a) Setting, (b) number of people, (c) text present, and (d) cut present. Bars represent standard errors of the mean across video clips.

Figure 4 shows that there are no clear effects of the various video aspects on the percentage of significant differences between groups. No interaction between politician and setting was found, *F*(2, 27) = 1.49, *p* = .24, η^2^ = 0.010. Neither were there significant main effects of setting, *F*(2, 27) = 0.34, *p* = .72, η^2^ = 0.024, or politician, *F*(1, 27) = 4.01, *p* = .055, η^2^ = 0.13. No interaction between the number of people and politician is found either, *F*(2, 36) = 1.47, *p* = .23, η^2^ = 0.039. Also here, the main effects of number of people, *F*(1, 36) = 2.41, *p* = .13, η^2^ = 0.063, and politician, *F*(1, 36) = 3.51, *p* = .069, η^2^ = 0.089, do not reach statistical significance. No significant interaction is found between the presence of text and politician, *F*(1,35) = 1.03, *p* = .32, η^2^ = 0.029, and no main effect of the presence of text is found, *F*(1, 35) = 1.72, *p* = .20, η^2^ = 0.047. This time a significant main effect of politician is found, *F*(1, 35) = 4.85, *p* = .034, η^2^ = 0.12. No interaction is found between politician and the presence of a cut in the video, *F*(1, 36) = 1.88, *p* = .18, η^2^ = 0.050. No main effects are found of the presence of a cut, *F*(1, 36) = 0.42, *p* = .52, or the politician, *F*(1, 36) = 3.02, *p* = .91, η^2^ = 0.077.

### Viewing tendencies

Earlier (Figure 1b), we saw that the distance to the group centres may fluctuate over time (e.g., for the fourth Corbyn video, the first section of the video appears to have smaller differences for the Labour participants, whereas later in the video, this difference is smaller for non-Labour participants). The videos shown in this illustration, however, had a relatively low percentage of significant differences and may therefore not reveal clear consistent differences between groups.

**Figure 5.**
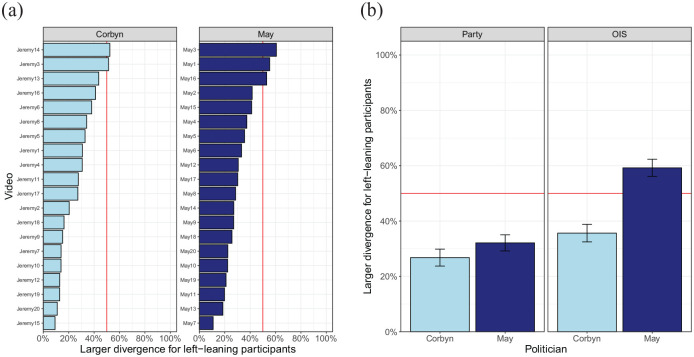
(a) Divergence per video for the party split. (b) Average divergence per politician and participant split.

To examine to which extent videos differ in the divergence in gaze position between the two groups, Figure 5 plots the proportion of frames with a larger divergence for left-leaning participants. This shows that for most videos, the party split yields less divergence in gaze position for the left-leaning participants. The only combination of participant split and politician for left-leaning participants do not systematically show a smaller divergence is the OIS split for videos of May.

**Figure 6.**
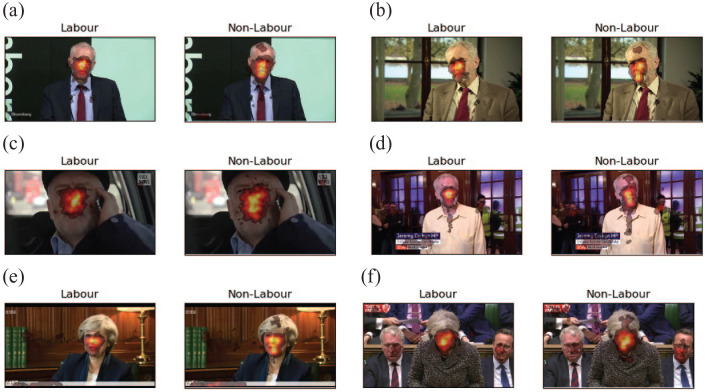
Heatmaps for videos with large differences between left-leaning participants and right-leaning participants (party split), suggesting that right-leaning participants more strongly focus on the mouth region. (a) Corbyn-7, (b) Corbyn-15, (c) Corbyn-18, (d) Corbyn-20, (e) May-7, and (f) May-13.

Although Figure 5 indicates a larger divergence of fixation locations in right-leaning participants, the images do not make clear whether such divergence is due to looking more at the background, or more towards different regions of the face of the politicians in the videos. To investigate this issue, Figure 6 plots a series of heatmaps (based on a party split of the participants) superimposed on a still from each video (in these videos, the scene was relatively constant). The heatmaps suggest that right-leaning participants more often fixate the mouth, compared with the left-leaning participants.

### Classifying unseen participants using machine learning

Ultimately, our method may be used to classify participants automatically into groups. To examine whether the proposed method may indeed play a role in saccade diagnostics (to “diagnose” group membership on the basis of eye movements), we make use of machine learning techniques, focusing on the split based on party affiliation (Labour vs. non-Labour), and the average distance to the group centre per video for the two U.K.-based politicians (Corbyn and May).

As explained in the “Method” section, we split the data into a training set and a test set, and only introduce the test set at the very last stage of the procedure. Performance on this test set of participants therefore mimics performance of a newly tested set of participants. Because of the relatively “small” number of participants (in terms of machine learning; for eye tracking purposes, we had a relatively normal size sample), we repeatedly split the data into training and test set to reduce the effects of the particular split of training and test set in the average data.

**Figure 7.**
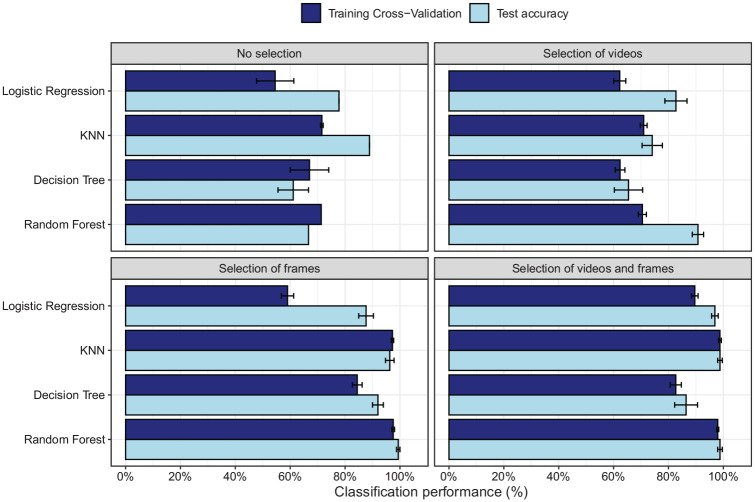
Accuracy on the training set (fivefold cross-validation accuracy) and test set for the four different machine learning models for selection of “significant” frames, videos (>10% “significant” differences), frames and videos, or no selection.

Figure 7 shows the prediction accuracy for the different models and the different types of input data (all videos/selection of videos, all frames/selection of frames) based on distances towards the group centres (results for horizontal and vertical distances between groups are shown in the Supplemental Material). As the two groups (Labour vs. non-Labour) were almost identical in size, we here focus on accuracy as the measure of performance of the models. For the training set, we plot the fivefold cross-validation accuracy, whereas for the test set, accuracy of the entire test sample is shown.

When all frames are used, prediction accuracy varies between chance level and around 80% (chance level = 52%, as 23 participants of 44 were non-Labour). Unexpectedly, the test set sometimes shows higher performance than the cross-validation of the training set. As no hyperparameter tuning was performed during training, the cross-validation therefore also reflects largely unseen data, although some leaking of information about group membership into the training data may occur when computing the distance to the group centres. The lower cross-validation performance may therefore reflect some level of underfitting. Differences between training and test accuracy are small for the KNN and random forests when selection of frames is applied, suggesting lower levels of under- or overfitting in these conditions while at the same time showing excellent group membership prediction.

The main improvement of performance is found after selection of frames. Selection of videos with more than 10% “significant” frames improves accuracy somewhat, but not to a large extent. Selection of videos may still be beneficial if the test would be adopted for group membership classification in a new sample, as it would reduce testing times (as fewer videos need to be presented).

As indicated, more pronounced improvement is found when “significant” frames are selected. For the KNN and random forest classifiers, performance even reaches almost perfect accuracy both on the training and the test set. Selection of frames thereby benefits prediction, but it will not reduce testing time, as just showing the selected frames will lead to fragmented videos.

When a selection is performed both on the frames and videos, a new group of participants tested on this smaller number of videos can be classified for political affiliation with an almost 100% accuracy (Figure 7d), just like when just frames are selected. This suggests that the selection of frames is what improves prediction accuracy. Selection of videos helps to reduce testing time, but has little effect on prediction accuracy.

**Figure 8.**
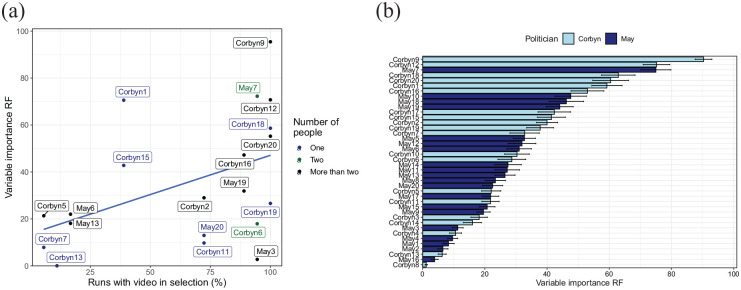
Variable importance for the random forest classifier. (a) Scatterplot showing the relation between the chance of a video being selected on the basis of the 10% “significant” frames criterion, and the variable importance of that video in the random forest prediction (when included). Videos shown on the right are almost always included, those on the left almost never (those never included are not shown in the plot). The dots are colour coded for the number of people in the video, showing no clear association between this aspect and the variable importance or chance of the video being included. (b) Variable importance of the various videos when all videos are included in the random forest prediction, colour coded for the politician shown. Videos of Corbyn had significantly higher variable importance than videos of May.

To examine whether particular videos more strongly contribute to the prediction of party affiliation, Figure 8 examines the variable importance of the best predicting model (the random forest classifier). Figure 8a shows that videos that were more likely to be selected on the basis of the 10% criterion also tended to have a stronger influence on the prediction. We then examined whether any of the features of the videos identified earlier (setting, number of people, text present, or cuts present) influenced variable importance when all videos were kept in the analysis (and the selection was based on frames within each video only). None of these features had a significant effect on the variable importance (Figure 8a colour codes the effect of the number of people in the video). One variable, namely, the politician shown (see Figure 8b), did have a significant effect: Videos of Corbyn had a significantly higher variable importance than videos of May, *F*(1, 178) = 31.9, *p* < .001. Earlier, we saw that Corbyn videos had larger percentages of “significant” frames for the party split that we are considering here. This again suggests a link between the number of “significant” frames and the importance for classification.

## Discussion

We here present a simple but effective data-driven method to examine group differences in eye movement patterns towards dynamic stimuli (video clips). Eye movement patterns for such stimuli have been notoriously difficult and labour-intensive to analyse, possibly discouraging researchers to use such stimuli although they are more ecologically valid than static images. Traditionally, top-down approaches, such as ROI analyses, have been used that require the definition of regions for each individual video frame. In such methods, however, it can be unclear what relevant ROIs are, particular in domains that involve stimuli other than people in normal settings (e.g., surgical images). Some data-driven methods, such as iMap (Caldara & Miellet, 2011), have been developed, but these may be difficult to adopt for users unfamiliar to running software under MATLAB. These methods also tend to be computationally expensive and it may therefore be difficult to extend their application from images to videos.

For our method, we adopted a data-driven approach where group differences are used to identify relevant video frames, relevant videos, and predicted group membership on the basis of these selections of frames and videos. In developing this method, we gave preferences to a method that is at its heart relatively simple because complex methods may hold researchers back in adopting the approach. We therefore used a widely used statistical test (Student’s *t*-test) to compare gaze positions for the two groups of interest. It is important to stress that the *t*-tests used do not provide conclusions about the statistical significance of the differences between groups, as multiple comparisons lead to an inflation of the Type I error when used without appropriate correction methods. In contrast to the iMap method (Caldara & Miellet, 2011), our method therefore does not provide an indication of the statistical significance of any observed differences.

We identified two methods to compare groups, one that examines differences in the central tendency of gaze position (by comparing horizontal and vertical gaze positions—results discussed in the Supplemental Material) and one that examines differences in variation in gaze position within groups (by comparing the distance to the group centres—results shown in the main text). Both methods predict group membership of unseen participants with a better than 90% accuracy, when either a KNN or random forest classifier is used after selection of frames with a “significant” group difference in the training set. Although heatmaps suggest that groups may differ in their focus on the mouth of the politician, the method that examines variation in gaze position outperformed the method examining gaze position differences. Restricting classification to the videos with a larger percentage of “significant” frames, but without selecting frames, had a weak effect on classification. Selection of videos may therefore reduce testing time, but does little for prediction.

The machine learning approach adopted here is fairly complex, but the actual method to identify relevant videos and relevant sections of videos does not require machine learning. Researchers can use the method by simply performing *t*-tests comparing groups for each frame of each video.

We have shown that the method can be used to isolate videos that have a large number of frames with group differences, and sections of videos that show larger differences. These videos and sections of videos can be used not only to better understand such group differences but also to refine eye movement tests to classify people into groups based on their eye movements by reducing the testing time. Although the method can also be used to select relevant video frames, presenting just those video frames in a sequence would make little sense, unless they occur in longer sequences. Selection of frames therefore serves mainly to improve classification performance.

Our findings add to earlier findings showing differences in gaze variability across observers (Dorr et al., 2010) by demonstrating differences in gaze variability between groups of participants. As indicated, the method also adds to earlier work on testing statistical differences between viewing patterns (Caldara & Miellet, 2011) by providing a computationally less expensive method that can be extended to videos. Our method is an exploratory approach: The aim is to uncover sections of videos with differences and videos with larger differences, rather than to test the statistical significance of these differences. The iMap method (Caldara & Miellet, 2011) may be used after identifying frames and videos with large group differences to statistically test the differences uncovered with our method.

Our method also adds to studies that showed differences in eye movements patterns during different tasks (Borji & Itti, 2014; DeAngelus & Pelz, 2009; Haji-Abolhassani & Clark, 2014). These previous studies and our method both present the same stimuli to participants, and as a consequence, any observed differences in eye movement patterns cannot be due to the stimuli. The difference is that these past studies have focused on differences that arise under different tasks (a within-subjects comparison), whereas the current application has focused on differences between groups of participants (a between-subjects comparison). Our method, however, can also be used to study the effect of task on viewing videos (with appropriate counterbalancing of the conditions) and can therefore also be used for within-subjects comparisons.

Our method extends the method introduced by Khan et al. (2012), but instead of comparing traces of pairs of observers, group differences are examined. Importantly, by identifying video clips with large significant differences between groups, our method can also aid in the identification of still images best suitable for detecting group differences if video playback is not an option.

We tried to determine what aspects of the videos were associated with group differences, but interestingly, none of the aspects considered was clearly associated with these differences. This was in contrast to our prediction that when only the politician would be in view (with little else to look at), smaller group differences would be found. Inspection of heatmaps of fixations for videos with larger differences between the two groups, based on a party split, suggested that right-leaning participants may fixate the mouth to a larger extent than left-leaning participants. Studies have suggested that observers with autism may focus less on the eyes region, although it is less clear whether this also leads to more fixations on the mouth (Klin et al., 2002; Papagiannopoulou et al., 2014).

This leads to a possible issue with our method: We cannot exclude the possibility that differences in viewing patterns between left- and right-leaning participants were exclusively due to party affiliation. Participants in the two groups may have differed in other ways, for example, on how they would have scored on an autism spectrum scale. As we did not anticipate any differences between the two groups in this respect, we did not administer a scale to test for differences on the autism spectrum. A follow-up study may provide more insight in whether the observed differences were solely due to political orientation. Such a study could also more systematically vary the various aspects of the videos to determine what drives the differences in viewing patterns with political orientation.

It is important to mention that our method is entirely data-driven: It does not make any assumptions about difference between groups, or reasons for such differences. In this respect, our method differs from other methods that are often considered to be data-driven, such as saliency models (Itti & Koch, 2000), but which, in fact, test assumptions about how the brain assigns priority to different features (e.g., colour, luminance, contrast) of an image.

To uncover sections of videos with “significant” (or near-“significant”) frames, run length detection may be used, which may merge near-significant frames with previous runs if the same parity of the difference is found. A run length analysis of significant left-larger, right-larger, and nonsignificant differences (results not shown) suggested that runs with significant differences were generally short. Isolating such runs therefore may aid mostly the interpretation of the observed group differences and may be of less value in reducing the length of the videos for saccade diagnostics (the resulting sections would simply be too short).

## Conclusion

For eye tracking research to move towards the use of more ecologically valid dynamic stimuli, new methods are needed to deal with the analysis of eye movement data for such stimuli. In this article, we present a simple but effective way to detect group differences for dynamic stimuli and select stimuli that are most informative of such group differences. We validated our method by predicting political affiliation based on eye movements towards video clips of videos. The method is easily extended to other domains, such as predicting psychological disorders or skill and expertise on the basis of people’s eye movements. Importantly, our method shows that running a pretest initially to determine which video sections and videos show different gaze directions in different groups, researchers can create a powerful diagnosis tool. We encourage others to utilise and expand on this research to develop robust ways to improve our understanding of eye movements towards dynamic stimuli.

## Supplemental Material

sj-docx-1-qjp-10.1177_17470218211048060 – Supplemental material for Data-driven group comparisons of eye fixations to dynamic stimuli Click here for additional data file. Supplemental material, sj-docx-1-qjp-10.1177_17470218211048060 for Data-driven group comparisons of eye fixations to dynamic stimuli by Tochukwu Onwuegbusi, Frouke Hermens and Todd Hogue in Quarterly Journal of Experimental Psychology

## Notes

1. The NSS is a metric that involves a saliency map and a set of fixations and aims to measure the saliency values at fixation locations along a subject’s scanpath (Le Meur & Baccino, 2013).
