# Supplementary material for: Data-driven group comparisons of eye fixations to dynamic stimuli
Source: Q J Exp Psychol (Hove). 2021 Sep 29;75(6):989–1003. doi: 10.1177/17470218211048060 (PMC9016662; doi:10.1177/17470218211048060)
Supplement: sj-docx-1-qjp-10.1177_17470218211048060 – Supplemental material for Data-driven group comparisons of eye fixations to dynamic stimuli [file sj-docx-1-qjp-10.1177_17470218211048060.docx]

Appendix

## Central tendency classification

In the main text, we found that group membership in a hold-out sample could be predicted with high accuracy on the basis of the distance to the group centres. This method may therefore reveal gaze avoidance behaviour that may be stronger in one group or another (e.g., non-Labour participants may try to avoid looking at the Labour politician). A more low-level, technical explanation, however, may be that participants in the non-Labour group may have had worse calibration of the eye tracker, leading to a larger variability in gaze position, and therefore a larger distance to the group centre.

Figure A1 therefore examines whether any systematic tendency towards larger distances to the group centre can be found in some participants (or all participants in the non-Labour group) that would reflect poor calibration. It shows that average distances towards the group centre varies substantially across videos. The broad distribution across videos tends to shifted towards lower values for Labour participants than for non-Labour participants, but the averages per video do not suggest poor calibration in specific participants or in the non-Labour group as a whole (i.e., no systematic shift of the distance towards the group centre for some or all non-Labour participants).


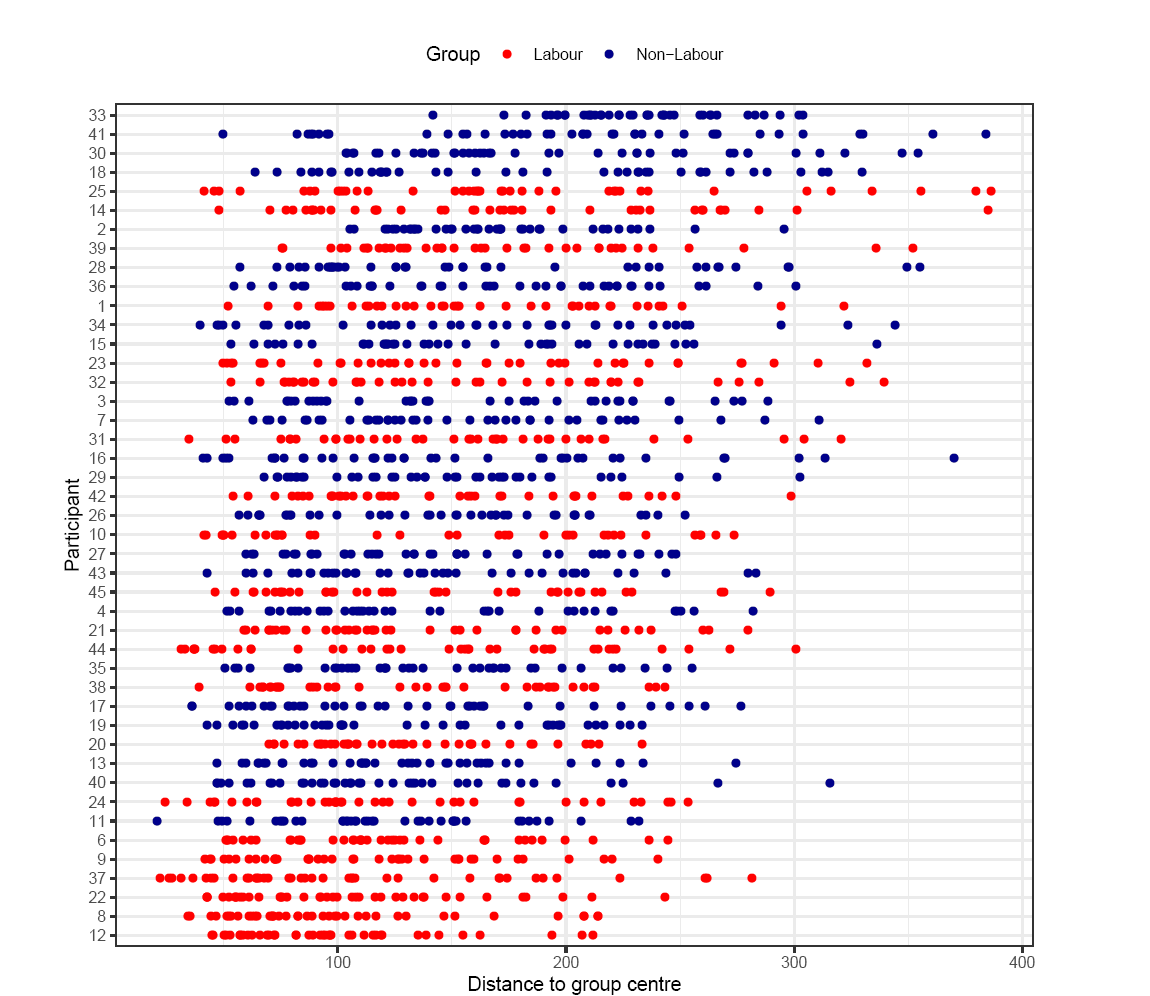


Figure A1: Distance to the group centre per video and participant. Participants with poor calibration are expected to have systematically large differences to their group centre across all videos (i.e., a cluster of dots to the right of the graph). Instead, a large variation in distances towards the centre is found within each participant, but this overall distribution tends to start at lower values for Labour participants.

The heatmaps of average gaze positions superimposed onto the images suggested that non-Labour participants may look at the mouth of the politician to a stronger extent than Labour participants. This could lead to a larger distance to the group centre (which could be in between the eyes and the mouth for non-Labour participants when gaze positions are equally distributed between eyes and mouth). A stronger focus on the mouth can also be expected to lead to group differences in the central tendency of gaze positions. It can therefore be expected that good group membership prediction should also be found for averages based on horizontal and vertical position, and filtering on the basis of differences in horizontal and vertical positions (rather than distance towards the group centres).


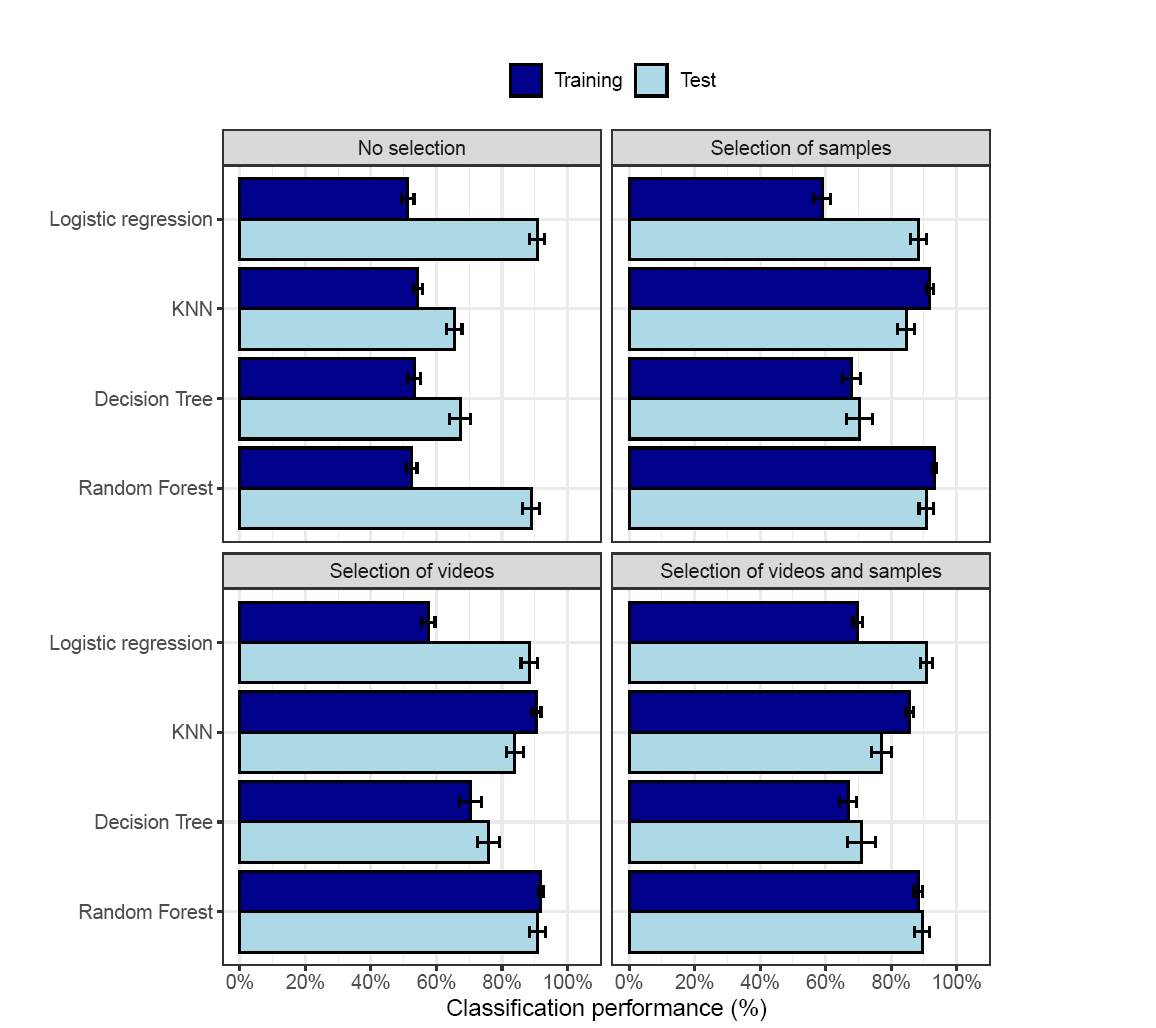


Figure A2: 5-fold cross-validation performance on the training set and accuracy on the (hold-out) test set for four classifiers on the basis of average horizontal and vertical gaze positions per participant, after possible filtering for videos, frames or videos and frames.

Figure A2 tests this prediction by examining prediction accuracy based on average horizontal and vertical gaze position, after (possible) filtering of frames of videos with large group differences in these gaze positions. It shows that group membership prediction in unseen data (either in the training set - based on cross-validation) or the test set (hold-out sample) is at around 90% for the KNN and random forest classifiers (selection of videos and/or samples). This is slightly worse than was found for distances towards the group centres, but still well above chance level. Again, we see much higher classification accuracy for the logistic regression on the test set than the training set, and these results therefore need to be interpreted with caution (while no hyperparameters are tuned during training, some information about group membership may leak into the training cross-validation, but not in the validation with the test set). For the KNN and random forest classifiers, training cross-validation accuracy and test set accuracy are similar, suggesting low levels of under- or overfitting.

To better understand how the selection of frames affects the average group location in each video, Figure A3 shows these group centres before and after filtering of the ‘significant’ frames. Each pair of dots shows the data for one video, with the red dot showing the group centre of Labour participants, and the blue dot the group centre of non-Labour participants. By focusing on just those video frames with ‘significant’ differences, the group centre differences are amplified, which facilitates the classification, but also prediction (unseen data) task, suggesting that something in these video frames has a different effect on Labour and non-Labour participants.


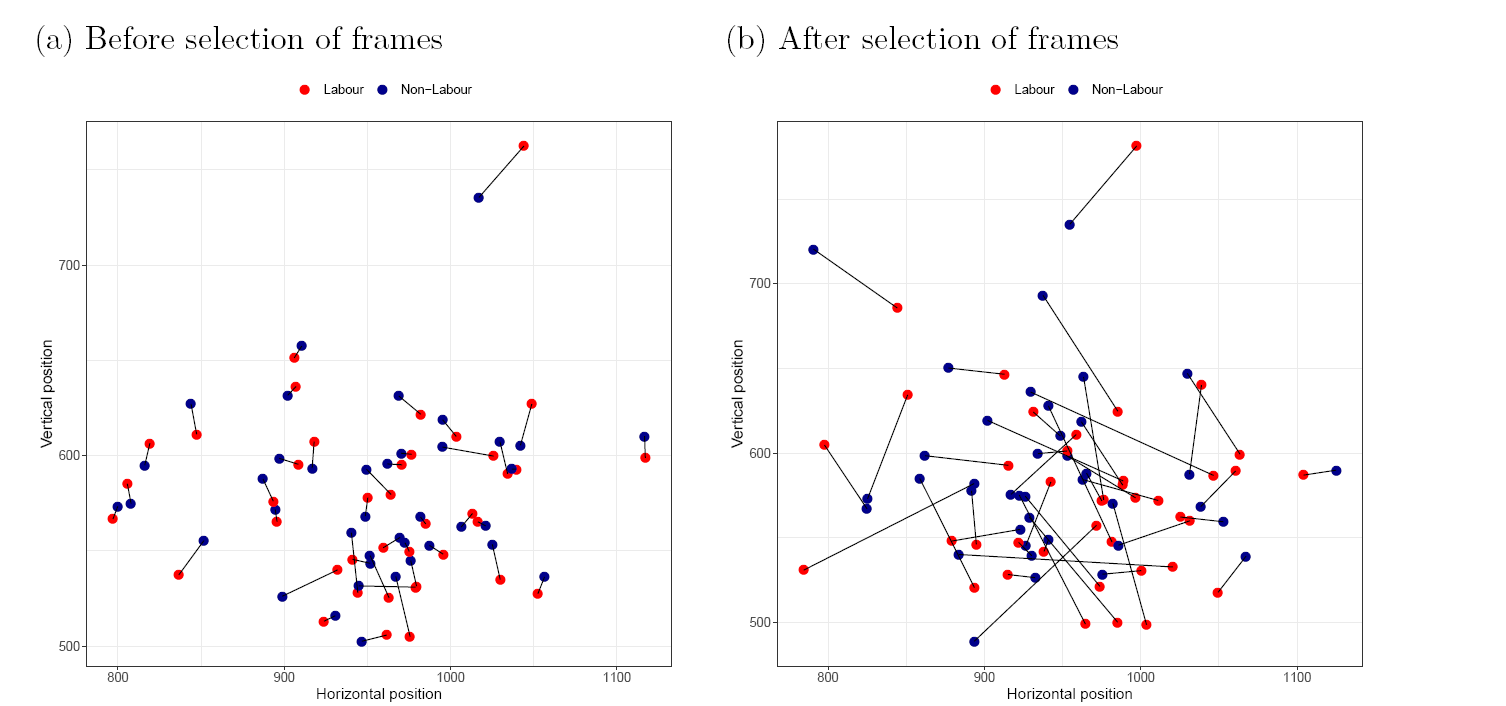


Figure A3: Group centres for each video for Labour and non-Labour participants before selection of ‘significant’ frames, and after selection. By focusing on ‘significant’ frames, average group centre differences are amplified.

To examine whether differences in central tendency and differences in variation of position show an association across videos, Figure A4 compares these two measures per video. This plot shows that videos that have a large group difference on one measure do not automatically have a large group difference on the other measure, suggesting that both measures appear to tap into distinct aspects of group differences (although both predictive of group membership).


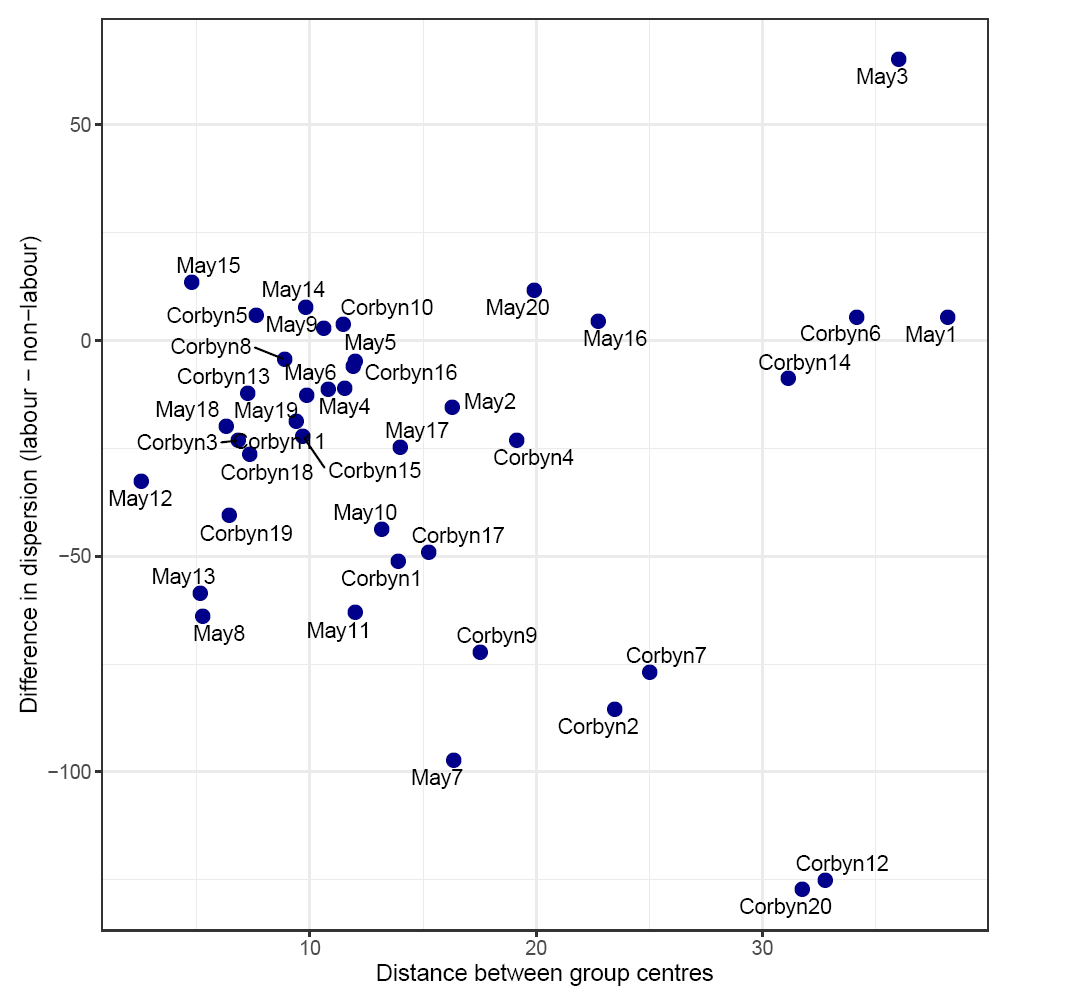


Figure A4: Comparison of group differences based on horizontal and vertical distances and on the basis of distance to the group centres, both after filtering of ‘significant’ frames. No clear association between the two methods is found ($r$ = -0.093, $p$ = 0.56), suggesting that differences in horizontal and vertical location (differences in central tendency) do not always coincide with large difference in distance to the group centre (avoidance differences).
